# Cellular Receptor Tyrosine Kinase Signaling Plays Important Roles in SARS-CoV-2 Infection

**DOI:** 10.3390/pathogens14040333

**Published:** 2025-03-31

**Authors:** Shania Sanchez, Brigitte H. Flannery, Hannah Murphy, Qinfeng Huang, Hinh Ly, Yuying Liang

**Affiliations:** Department of Veterinary and Biomedical Sciences, College of Veterinary Medicine, University of Minnesota, Saint Paul, MN 55108, USA; sanch737@umn.edu (S.S.); flann103@umn.edu (B.H.F.); murp1625@umn.edu (H.M.); huangq@umn.edu (Q.H.); hly@umn.edu (H.L.)

**Keywords:** SARS-CoV-2, antiviral therapeutic, receptor tyrosine kinase (RTK), host signaling

## Abstract

Current antiviral treatments often target specific viral components, which can lead to the rapid emergence of drug-resistant mutants. Targeting host signaling pathways, including their associated cellular factors, that are important for virus replication is a novel approach toward the development of next-generation antivirals to overcome drug resistance. Various cellular receptor tyrosine kinases (RTKs) have previously been shown to play important roles in mediating viral replication including coronaviruses. In this study, we examined the roles of RTKs in SARS-CoV-2 replication in two cell lines, A549-ACE2 (human lung epithelial cells) and Vero-E6 (African Green Monkey kidney cell), via chemical inhibitors. We showed that the HER2 inhibitor Lapatinib significantly reduced viral replication in both cell lines, the TrkA inhibitor GW441756 was effective only in A549-ACE2 cells, while the EGFR inhibitor Gefitinib had little effect in either cell line. Lapatinib and GW441756 exhibited a high therapeutic index (CC_50_/EC_50_ > 10) in A549-ACE2 cells. Time-of-addition experiments indicated that Lapatinib may inhibit the early entry step, whereas GW441756 can affect post-entry steps of the viral life cycle. These findings suggest the important roles of HER2 and TrkA signaling in SARS-CoV-2 infection in human lung epithelial cells and support further investigation of RTK inhibitors as potential COVID-19 treatments.

## 1. Introduction

Coronaviruses can infect both humans and animals [1]. Some human coronaviruses can cause severe and deadly respiratory diseases, such as the severe acute respiratory syndrome virus (SARS-CoV) causing an outbreak in 2002–2003, the Middle East respiratory syndrome coronavirus (MERS-CoV) causing epidemics since 2012, and SARS-CoV-2 causing the COVID-19 pandemic in 2019–2023 [2]. The COVID-19 disease can range from asymptomatic or mild respiratory disease to severe lung injury, such as pneumonia or acute respiratory syndrome (ARS) that can lead to multi-organ failure and death [3]. Although SARS-CoV-2 vaccines are available, SARS-CoV-2 continues to circulate in humans, with emerging variants evolving to evade vaccine-induced immunity [4].

Currently, there are four FDA-approved drugs for treating SARS-CoV-2 infections. These include two virus-targeted antivirals, Veklury (remdesivir) and Paxlovid (nirmatrelvir and ritonavir), and two immunomodulator drugs, Oluminant (baricitinib) and Actemra (tocilizumab) [5]. Veklury targets the viral RNA-dependent RNA polymerase and is approved for use in adults and children, whereas Paxlovid targets the viral main protease Mpro and is approved for use in adults only. Oluminant is a JAK kinase inhibitor, and Actemra is an IL-6 inhibitor, both for use only in hospitalized adults [5]. Under specific conditions, drugs may be prescribed under Emergency Use Authorization (EUA), such as Lagevrio (molnupiravir), an RNA nucleoside analog that inhibits viral replication through the induction of lethal mutagenesis [6,7].

Antiviral therapeutics typically target viral components, which can lead to the emergence of drug-resistant variants, reducing their effectiveness over time. Targeting host factors important for viral replication offers an attractive alternative approach to reduce the chances of drug resistance [8]. Receptor tyrosine kinases (RTKs) are a group of cell surface receptors that, upon ligand binding, undergo dimerization and autophosphorylation to trigger downstream signaling pathways, like the Ras/ERK/MAPK, PI3K/Akt, and JAK/STAT pathways [9]. RTKs mediate important cellular functions, such as growth, metabolism, survival, migration, and differentiation [9]. Previous studies have shown that viruses utilize the active RTK signaling pathways to promote their own replication, such as the influenza virus and coronaviruses [10,11]. For example, the transmissible gastroenteritis virus (TGEV) spike protein binding to the epidermal growth factor receptor (EGFR) has been shown to aid viral entry [12]. MERS-CoV replication has been shown to be inhibited by suppressing MAPK/ERK and PI3K/AKT/mTOR signaling [13]. The role of RTK signaling in SARS-CoV-2 replication is not well understood.

In this study, we evaluated the roles of selective RTKs in SARS-CoV-2 replication in vitro, using small molecule inhibitors known to target human epidermal growth factor receptor 2 (HER2), tropomyosin kinase receptor A (TrkA), and epidermal growth factor (EGFR). We showed that the HER2 and TrkA inhibitors can significantly reduce SARS-CoV-2 replication in human lung epithelial cells, with a high therapeutic index value, and they act on different stages of the viral life cycle. Our study suggests that RTKs, such as HER2 and TrkA, may play distinct roles in SARS-CoV-2 replication, which can be explored as potential antiviral therapeutic targets.

## 2. Materials and Methods

### 2.1. Cells, Viruses, and Compounds

Vero-E6 cells (African Green Monkey kidney epithelial cells) were obtained from ATCC (#CRL-1586). A549-ACE2 cells (human lung epithelial cells stably expressing human ACE2) were obtained from BEI resources (NR-53522). BHK-21-ACE2 cells (baby hamster kidney fibroblast cells stably expressing human ACE2) were obtained from Dr. Guangxiang Luo (University of Alabama at Birmingham). These cells were grown in Dulbecco’s modified Eagle’s medium supplemented with 10% fetal bovine serum and 1% penicillin/streptomycin (Gibco^TM^,15070063, ThermoFisher Scientific, Waltham, MA, USA) at 37 °C in humid air with 5% CO_2_. SARS-CoV-2 virus strain (WA1) and SARS-CoV-2-nLUC virus were obtained from BEI resources (NR-54003) and used in Biosafety level 3 (BSL-3) facilities. SARS-CoV-2 (WA1) was cultivated in Vero-E6 cells, and its titer was determined by a plaque assay on BHK-21-ACE2 cells. SARS-CoV-2-nLUC virus was cultivated in BHK-21-ACE2 cells, and its titer was determined by a plaque assay on BHK-21 ACE2. Four small molecule inhibitors were used in this study (AG879, Lapatinib, Gefitinib, and GW441756). AG879 (MedChemExpress, HY-20878, Monmouth Junction, NJ, USA) is a selective inhibitor of both tropomyosin kinase receptor A (TrkA) and human epidermal growth factor receptor 2 (HER2), Lapatinib (Sigma Aldrich, CDS022971, St. Louis, MO, USA) is a reversible inhibitor of both epidermal growth factor receptor (EGFR) and HER2, Gefitinib (Sigma Aldrich, SML1657) is a selective inhibitor of EGFR, and GW441756 (MedChemExpress, HY-18314) is a selective inhibitor of TrkA [14,15,16].

### 2.2. RNA Extraction and Quantitative RT-PCR (RT-qPCR)

A total of 400 μL of culture fluids was mixed with 800 μL of DNA/RNA shield (Zymo Research, R2141, Irvine, CA, USA). Viral RNAs were extracted per the manufacturer’s instructions. The extracted RNAs were dissolved in 50 μL of nuclease-free water. One-step quantitative reverse-transcribed PCR (RT-qPCR) using the Luna Universal One-Step RT-qPCR Kit (New England Biolabs, E3005L, Ipswich, MA, USA) was performed on the extracted RNA samples in duplicates using primers specific for the envelope (E) regions of the SARS-CoV-2 genome.

### 2.3. Western Blot

A549-ACE2 cells in 10 cm^2^ plates were infected with SARS-CoV-2 (WA1) at MOI = 1. After virus infection, the cells were rinsed in cold phosphate-buffered saline (PBS) and lysed in a radioimmunoprecipitation assay (RIPA) buffer containing phosphate inhibitor cocktail #3, Sodium Orthovanadate, Pepstatin, Leupeptin, Phenylmethylsulfonyl fluoride, and Aprotinin. After centrifugation (15 min, 12,000× *g*, +4 °C), protein loading dye with 5% beta-mercaptoethanol was added, and the protein was boiled for 15 min to denature. Total protein was subjected to 8% SDS-PAGE gel, blocked with 5% BSA, and then blotted onto a nitrocellulose membrane (Thermo Scientific, #88018, Waltham, MA, USA) using the trans-turbo semi-dry transfer apparatus (Bio-Rad, Hercules, CA, USA). The following antibodies were used for immunoblotting: rabbit anti-human TrkA polyclonal antibody (Cell Signaling Technology, 2505S, Danvers, MA, USA), rabbit anti-human pTrkA monoclonal antibody (mAb) (Cell Signaling Tech, 4168S), rabbit anti-human HER2 mAb (Cell Signaling Tech, 2165S), rabbit anti-human pHER2 mAb (Cell Signaling Tech, 2243L), mouse anti-human EGFR mAb (Cell Signaling Tech, 2239S), rabbit anti-human pEGFR mAb (Cell Signaling Tech, 3777S), rabbit anti-human GAPDH mAb (Cell Signaling Tech, 2118S), anti-rabbit HRP conjugated (Bio Rad, 1706515, Hercules, CA, USA), and anti-mouse IgG HRP conjugated (R&D, HAF007). After immunoblotting, the membrane was applied with Immobilon Western Chemiluminescent HRP Substrate (MilliporeSigma, WBULP, Burlington, MA, USA) for imaging on an iBright FL1500 system (Thermo Fisher Scientific). Protein band intensities were quantified using Sciugo software 2.0.1 and normalized to the baseline protein levels in mock-infected cells.

### 2.4. Inhibition of SARS-CoV-2-nLUC Infection

A549-ACE2 cells (35,000 cells per well) seeded in 96-well plates were infected with SARS-CoV-2-nLUC virus at an MOI = 75. Three-fold dilutions of the respective RTK chemical inhibitors were added at different time points (−1, 0, 2, 4, 8, and 24 h). The cells were lysed at 24 h post-infection (hpi) with the addition of Nano luciferase substrate (Promega, #N1110, Madison, WI, USA). Luciferase signals were measured using the BioTek Synergy^TM^ 2 microplate reader (Agilent Technologies Inc., Santa Clara, CA, USA). The 50% effective concentration (EC_50_) for each inhibitor was calculated using GraphPad Prism 10.4.1.

### 2.5. MTT Assay

Cells in 96-well plates were treated with 3-fold dilutions of RTK inhibitors or dimethyl sulfoxide (DMSO) (Sigma Aldrich, D2650) as a control in duplicates in a total of 100 μL growth medium for 24 h. A total of 10 μL of freshly made MTT (3-[4,5-dimethylthiazol-2-yl]-2,5- diphenyltetrazolium bromide) labeling reagent (final concentration 0.5 mg/mL) was added into each well, and the cells were incubated at 37 °C for 4 h. After incubation, 100 μL of solubilization buffer (Cell Proliferation Kit I MTT, Roche #11465007001, MilliporeSigma, Burlington, MA, USA) was added to each well to dissolve the purple formazan crystals, and the plates were incubated at 37 °C overnight. The spectrophotometric absorbance of the MTT signals was measured at an absorbance of 570 nm. The 50% cytotoxic concentration (CC_50_) was calculated as the concentration needed to reduce cellular viability to 50%.

### 2.6. Statistical Analysis

Statistical comparison of the different treatment groups throughout the manuscript was performed in GraphPad Prism using Student’s *t*-test. *, *p*< 0.05; **, *p* < 0.01; ***, *p* < 0.001.

## 3. Results and Discussion

The SARS-CoV-2 inhibition assays were conducted in triplicates across at least two independent experiments in A549-ACE2 and Vero-E6 cells using four chemical inhibitors known to target certain RTKs: AG879, Lapatinib, Gefitinib, and GW441756. AG879 is a selective inhibitor of both tropomyosin kinase receptor A (TrkA) and human epidermal growth factor receptor 2 (HER2), whereas Lapatinib is a reversible inhibitor of both epidermal growth factor receptor (EGFR) and HER2, Gefitinib is a selective inhibitor of EGFR, and GW441756 is a selective inhibitor of TrkA [14,15,16]. We selected these four chemical inhibitors because they are established inhibitors of respiratory viruses, including Influenza A virus (IAV). These RTKs are also known to be present in lung epithelial cells and have previously been utilized as therapeutic agents in the treatment of lung cancer [17].

**Figure 1:**
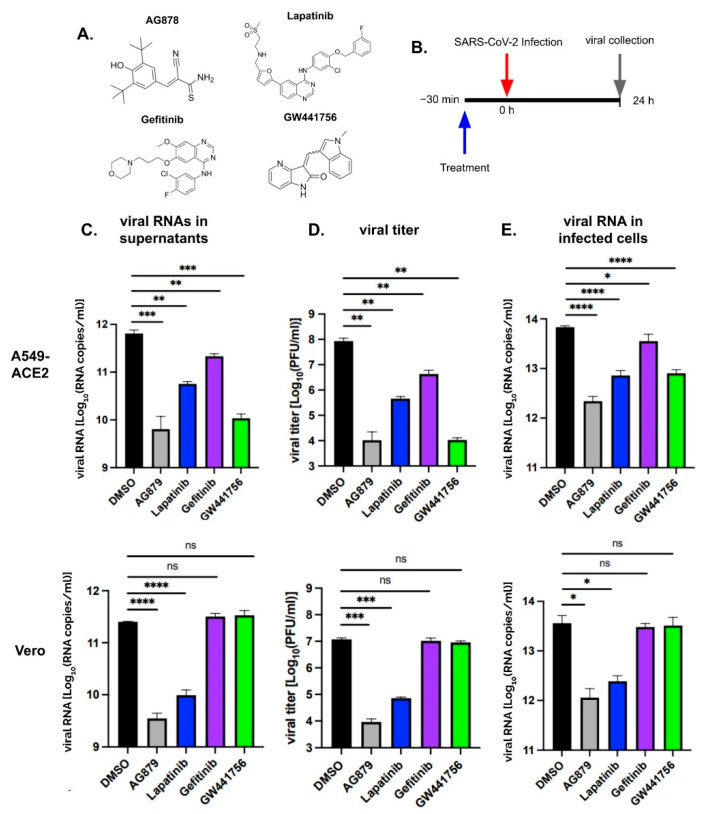
Chemical structures of RTK inhibitors AG879, Lapatinib, Gefitinib, and GW441756 (**A**). Timeline of infection, treatment, and viral collection (**B**). SARS-CoV-2 replication in cells pre-treated with RTK inhibitors. A549-ACE2 (top panels) and Vero-E6 (bottom panels) were treated for 30 min and then infected with SARS-CoV-2 (WA1) at an MOI = 0.01. Supernatants were collected, and viral particles were quantified by measuring viral RNA via RT-qPCR (**C**) and infectious particles via a plaque assay (**D**). Viral RNA levels in the infected cells were quantified by RT-qPCR (**E**). ns, *p* > 0.05; *, *p* < 0.05; **, *p* < 0.01; ***, *p* < 0.001; ****, *p* < 0.0001.

We first treated cells 30 min prior to SARS-CoV-2 (WA1 strain) infection at an MOI = 0.01 for 24 h with 10 µM of respective inhibitors or with 0.1% DMSO as a negative control. The amounts of the released viral particles in the cell supernatants were quantified by RT-qPCR and shown as viral RNA copy number per ml in the log scale (Figure 1C). In the A549-ACE2 cells (top panel), all four inhibitors significantly reduced viral particles when compared to DMSO. The reduction was 2 logs with AG879 and GW441756 treatment, 1 log with Lapatinib, and 0.5 log with Gefitinib. In the Vero-E6 cells (bottom panel), however, only AG879 and Lapatinib caused a significant reduction in viral particles by 1.5–2 logs, while Gefitinib and GW441756 showed no evidence of viral inhibition. Thus, AG879 and Lapatinib strongly inhibit SARS-Co-2 production in both cell lines, while GW441756 shows strong inhibition only in A549-ACE2 cells, and Gefitinib exhibits mild inhibition in A549-ACE2 cells.

As the amount of viral RNA does not always correlate with the number of infectious particles, we validated the results by measuring infectious virus via a plaque assay and showed the results as plaque forming units (PFU) per ml in the log scale (Figure 1D). Similar results as the viral RNA levels were observed for infectious viral titers. In the A549-ACE2 cells (top panel), all inhibitors significantly reduced infectious virus production when compared to DMSO. AG879 and GW441756 caused the strongest inhibition, reducing viral titers by 4 logs, followed by Lapatinib by 2 logs and Gefitinib by 1 log. In the Vero-E6 cells (bottom panel), only AG879 and Lapatinib significantly reduced infectious viruses by 2 to 3 logs, while Gefitinib and GW44175 did not reduce virus titers at all. Thus, from both viral RNAs and viral titer quantification, AG879 and Lapatinib can effectively inhibit SARS-CoV-2 replication in both cell lines, GW44175 is a strong inhibitor only in A549-ACE2 cells, while Gefitinib does not show strong inhibition in either cell line.

We also quantified the viral RNA levels in the infected cells by RT-qPCR (Figure 1E). A similar pattern of inhibition was observed as above for viral particle production. In the A549-ACE2 cells (top panel), AG879, Lapatinib, and GW441756 reduced viral RNAs by 1–2 logs, while Gefitinib only slightly reduced viral RNAs when compared to DMSO. In the Vero-E6 cells (bottom panel), only AG879 and Lapatinib significantly inhibited viral RNAs in the cells by 1–2 logs when compared to DMSO, while Gefitinib and GW441756 did not show any noticeable reductions. The correlated reduction in viral RNAs in the infected cells, viral RNAs in the supernatants, and viral titers for all four inhibitors suggests that these inhibitors may function at or before the stage of viral RNA synthesis. 

**Figure 2:**
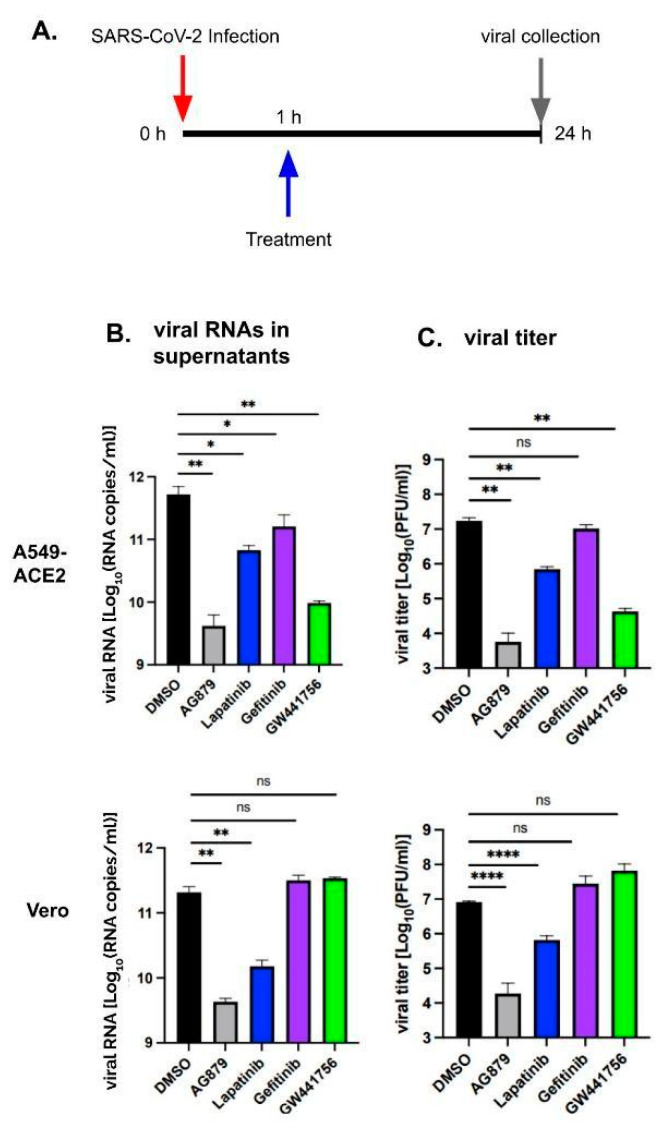
Timeline of infection, treatment, and viral collection (**A**). SARS-CoV-2 replication in cells treated with inhibitors 1 h post-infection (hpi). A549-ACE2 (top panels) and Vero-E6 (bottom panels) cells were infected with SARS-CoV-2 WA1 at an MOI = 0.01 and then treated with either DMSO or respective inhibitors at 1 hpi. Supernatants were collected, and viral particles were quantified by measuring viral RNA via RT-qPCR (**B**) and infectious particles via a plaque assay (**C**). ns, *p* > 0.05; *, *p* < 0.05; **, *p* < 0.01; ****, *p* < 0.0001.

We then infected cells with SARS-CoV-2 at a multiplicity of infection (MOI) of 0.01, followed by treatment with either DMSO or the respective inhibitors at 1 hpi. Viral RNA and infectious virus were then isolated 24 hpi. Viral particle levels were quantified using RT-qPCR (Figure 2B) and plaque assays (Figure 2C). In the A549-ACE2 cells (top panels), AG879 and GW441756 showed the strongest inhibition, decreasing the viral RNA level by 2 logs and viral titers by 2–3 logs. Lapatinib reduced viral RNAs and viral titers by 1 log, while Gefitinib showed a minor inhibition of viral RNAs (0.5 log) and no inhibition of viral titer. In the Vero-E6 cells (bottom panels), only AG879 and Lapatinib significantly inhibited viral production, reducing viral RNA levels by 1.5 to 2 logs and viral titers by 1 to 3 logs, whereas Gefitinib and GW441756 showed no reduction in either viral RNA or PFU. Notably, viral replication was inhibited to a similar extent when RTK inhibitors were administered before infection (Figure 1). These findings indicate that RTK inhibitors remain effective when applied post-SARS-CoV-2 infection.

To better understand at which step(s) during the SARS-CoV-2 life cycle the HER2 and TrkA inhibitors are effective in A549-ACE2 cells, we performed a time-of-addition (TOA) assay using SARS-CoV-2-nLUC, a replication-competent virus expressing the nLUC reporter gene. The A549-ACE2 cells were infected with SARS-CoV-2-nLUC at a high MOI and treated with the selective RTK inhibitors in serial dilutions at various time points from −1 to 24 hpi. The nLUC activity was measured at 24 hpi. The half maximal effective concentration (EC_50_) was calculated for each inhibitor at different time points (Table 1). Gefitinib was included as a negative control and exhibited no significant antiviral activity at any time points. The HER2 inhibitor Lapatinib was effective at low concentrations during early time points, and its efficacy declined (indicated by an increasing EC_50_) as time progressed, suggesting that it likely inhibits early viral entry steps potentially targeting processes such as viral attachment, endocytosis, and membrane fusion. Consistent with our findings, Lapatinib has been shown to inhibit SARS-CoV-2 viral entry in Calu-3 cells, another human lung epithelial cell line [18,19]. TrkA inhibitor GW441756, however, was shown to be similarly effective throughout the early and late time points (−1 to 8 hpi), suggesting that it acts at later post-entry steps in the virus life cycle, such as viral RNA replication and transcription, viral protein translation, and intracellular trafficking [20,21].

To evaluate the potential of Lapatinib, Gefitinib, and GW441756 as SARS-CoV-2 antiviral therapeutics, we calculated their therapeutic index (TI) value, defined as the 50% cytotoxic concentration (CC_50_) value divided by the EC_50_ value, in A549-ACE2 cells (Table 2). The CC_50_ value measured by the MTT assay and the EC_50_ value used at −1 hpi are shown in Table 1. Gefitinib was included as a negative control and exhibited no significant TI. Both Lapatinib and GW441756 exhibited a high TI value of >11, supporting further investigation of their potential usage in SARS-CoV-2 antiviral treatment. The dose–response inhibition curves for Lapatinib and GW441756 are shown in Supplementary Figure S1.

**Figure 3:**
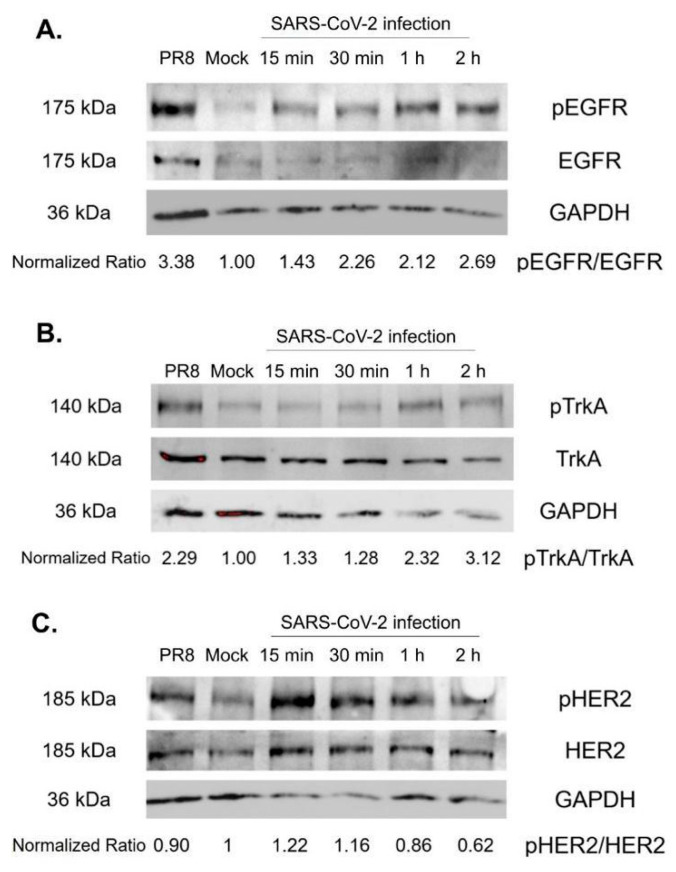
Western blot analysis of RTK activation in A549-ACE2 cells after SARS-CoV-2 infection. Cell lysates from mock infection (Mock), A/PR8 (PR8), and SARS-CoV-2 infection at different time points were analyzed by Western blotting to detect active and total forms of EGFR (**A**), TrkA (**B**), and HER2 (**C**). Protein bands were quantified using Sciugo software 2.0.1. The ratio of the phosphorylated form to total form was calculated and compared to the mock (set as 1.00).

To determine whether SARS-CoV-2 infection can activate the RTK pathways, A549-ACE2 cells were infected with SARS-CoV-2 (WA1 strain) at an MOI = 1, and cell lysates were collected at different time intervals from 0 to 2 h after infection. Cell lysates from the mock infection and from influenza A virus (IAV) A/PR8 infection (MOI = 1) were included as controls. IAV has been previously shown to activate all three RTKs [10,17,22]. Western blot analysis was performed with respective antibodies to detect the phosphorylated and total forms of EGFR (Figure 3A), TrkA (Figure 3B), and HER2 (Figure 3C). The ratio of active to total protein for each cell lysate was compared to the mock infection (set as 1) and shown at the bottom of the gel. Both EGFR and TrkA were further activated by A/PR8 and SARS-CoV-2 infections. The pHER2/HER2 ratio, however, was not significantly increased by either A/PR8 or SARS-CoV-2 infection, likely due to the high baseline level in the uninfected A549-ACE2 cells. These results suggest that SARS-CoV-2 infection can further upregulate RTK signaling pathways, though the biological significance of RTKs in viral infection needs to be individually evaluated.

Our study suggests that the HER2 and TrkA signaling pathways may play significant roles in SARS-CoV-2 infection in human lung epithelial cells and that targeting these pathways could serve as a potential antiviral therapeutic strategy. The data from this study and another study [19] indicate that HER2 may contribute to SARS-CoV-2 viral entry. Inhibition of HER2 has also been shown to significantly reduce infection by the alphacoronavirus swine acute diarrhea syndrome coronavirus (SADS-CoV) in vitro [23]. Ebola virus has been reported to utilize the HER2–PI3K–AKT signaling pathway to mediate virus uptake into cells [24]. TrkA has been implicated in both viral replication and lung inflammation during infection by influenza A virus and respiratory syncytial virus [22,25], with TrkA inhibition shown to block viral replication and reduce lung injury. Our data suggest TrkA signaling may impact post-entry steps of SARS-CoV-2 in human lung epithelial cells. Future studies are needed to determine the specific mechanism of action (MOA) for HER2 and TrkA in different cell types and to evaluate the effects of their inhibition in vivo. Expanding our understanding of RTK signaling pathways and their roles in viral replication can aid in the development of more effective broad-spectrum, host-targeted antiviral therapies.

## Figures and Tables

**Table 1:** The EC_50_ values of the RTK inhibitors in the TOA assay in A549-ACE2 cells.

EC_50_ (µM)	−1 h	0 h	2 h	4 h	8 h	24 h
**Lapatinib**	5.4	6.9	8	10.9	12.9	NI
**Gefitinib**	>100	>100	NI	NI	NI	NI
**GW441756**	10.1	12.4	12.1	11.2	13.5	NI

NI, no inhibition; EC_50_, half maximal effective concentration; h, hour.

**Table 2:** Therapeutic index (TI) for Lapatinib and GW441756 in A549-ACE2 cells.

	CC_50_ (µM)	EC_50_ (µM)	TI
**Lapatinib**	>100	5.4	>18.5
**Gefitinib**	>100	>100	1
**GW441756**	113.6	10.1	11.2

EC_50_, half maximal effective concentration; CC_50_, 50% cytotoxicity concentration; TI, therapeutic index.

## Data Availability

The original contributions presented in this study are included in the article/Supplementary Material. Further inquiries can be directed to the corresponding authors.

## Supplementary Materials

The following supporting information can be downloaded at: https://www.mdpi.com/article/10.3390/pathogens14040333/s1, Figure S1: Dose-response inhibition curves for Lapatinib and GW441756.

## Abbreviations

The following abbreviations are used in this manuscript:

CC_50_	50% cytotoxic concentration
EC_50_	50% effective concentration
EGFR	epidermal growth factor receptor
HER2	human epidermal growth factor receptor 2
hpi	hours post-infection
MOA	mechanism of action
MOI	multiplicity of infection
PFU	plaque forming units
SARS-CoV-2	severe acute respiratory syndrome coronavirus 2
TI	therapeutic index
TrkA	tropomyosin receptor kinase A
