# Supplementary figures and images for: Cellular Receptor Tyrosine Kinase Signaling Plays Important Roles in SARS-CoV-2 Infection
Source: Pathogens. 2025 Mar 31;14(4):333. doi: 10.3390/pathogens14040333 (PMC12030552; doi:10.3390/pathogens14040333)

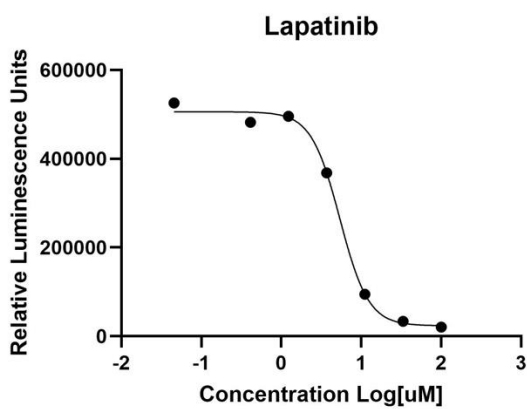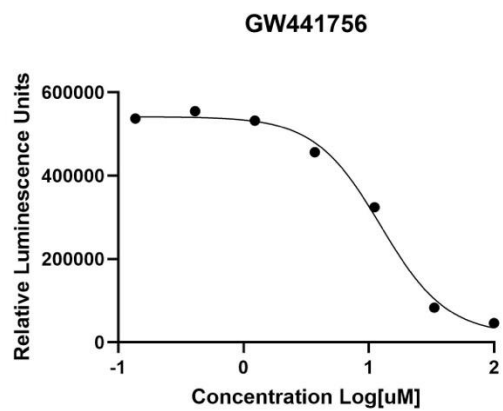

**Figure S1.** Dose-response inhibition curves for Lapatinib and GW441756

Supplement: Supplementary file 1 [file pathogens-14-00333-s001.zip › pathogens-3536713-supplementary.pdf]
